# Combination therapies augment the anti-tumor activity of agonist CD27 mAb in human CD27 transgenic mouse models

**DOI:** 10.1186/2051-1426-1-S1-P76

**Published:** 2013-11-07

**Authors:** Li-Zhen He, Lawrence J Thomas, James Testa, Jeffrey Weidlick, Crystal Sisson, Russell Hammond, Laura Vitale, Henry Marsh, Tibor Keler

**Affiliations:** 1 Celldex Therapeutics, Inc., Phillipsburg, NJ, USA; 2 Celldex Therapeutics, Inc., Needham, NJ, USA

## 

CDX-1127 is a fully human antibody to CD27, a TNF receptor superfamily member expressed on the majority of T cells and subsets of NK cells and B cells. We have previously characterized the co-stimulatory activities of CDX-1127 with human T cell cultures and a human CD27 transgenic mouse model (hCD27-Tg). Similar to the findings originally shown by M.J. Glennie and colleagues using an agonist anti-mouse CD27 mAb, CDX-1127 has potent antitumor activity as monotherapy in several syngeneic tumor models in hCD27-Tg mice. In the current studies, we sought to enhance the anti-tumor efficacy of CDX-1127 in challenging tumor settings by combination with clinically relevant therapies. Specifically, we focused on therapies that could decrease or control tumor growth while providing a source of antigen to drive anti-tumor immunity (e.g. chemotherapy or targeted therapy) and immune modifiers that may allow the CD27 driven T cell response to overcome self-regulation (e.g. checkpoint inhibitors or immune activators). In the EG7 delayed treatment model (average tumor size of ~ 50 mm3 when treatment initiated), the combination of CDX-1127 with cyclophosphamide significantly improved survival (>70% survival) compared to either agent alone (<30 % survival). Notably, we found that the combination therapy was associated with increases in the ratio of effector to regulatory T cells in the tumors compared to either single agent group. Additional combination studies with various agents are on-going, and initial studies with CDX-1127 combined with anti-CTLA-4 mAb has shown superior anti-tumor activity (median survival 36.5 days in combination versus 20 days with either single agent). These studies, along with the good safety profile of CDX-1127 reported in a Phase 1 clinical trial, supports the design of future combination studies in patients with cancer.

